# Translation Directed by Hepatitis A Virus IRES in the Absence of Active eIF4F Complex and eIF2

**DOI:** 10.1371/journal.pone.0052065

**Published:** 2012-12-18

**Authors:** Natalia Redondo, Miguel Angel Sanz, Jutta Steinberger, Tim Skern, Yuri Kusov, Luis Carrasco

**Affiliations:** 1 Centro de Biologia Molecular Severo Ochoa (Consejo Superior de Investigaciones Científicas-Universidad Autónoma de Madrid), Madrid, Spain; 2 Max F. Perutz Laboratories, Medical University of Vienna, Vienna, Austria; 3 Institute of Biochemistry, Center for Structural and Cell Biology in Medicine, University of Lübeck, Lübeck, Germany; 4 German Centre for Infectious Research, University of Lübeck, Lübeck, Germany; University of British Columbia, Canada

## Abstract

Translation directed by several picornavirus IRES elements can usually take place after cleavage of eIF4G by picornavirus proteases 2A^pro^ or L^pro^. The hepatitis A virus (HAV) IRES is thought to be an exception to this rule because it requires intact eIF4F complex for translation. In line with previous results we report that poliovirus (PV) 2A^pro^ strongly blocks protein synthesis directed by HAV IRES. However, in contrast to previous findings we now demonstrate that eIF4G cleavage by foot-and-mouth disease virus (FMDV) L^pro^ strongly stimulates HAV IRES-driven translation. Thus, this is the first observation that 2A^pro^ and L^pro^ exhibit opposite effects to what was previously thought to be the case in HAV IRES. This effect has been observed both in hamster BHK and human hepatoma Huh7 cells. In addition, this stimulation of translation is also observed in cell free systems after addition of purified L^pro^. Notably, in presence of this FMDV protease, translation directed by HAV IRES takes place when eIF2α has been inactivated by phosphorylation. Our present findings clearly demonstrate that protein synthesis directed by HAV IRES can occur when eIF4G has been cleaved and after inactivation of eIF2. Therefore, translation directed by HAV IRES without intact eIF4G and active eIF2 is similar to that observed with other picornavirus IRESs.

## Introduction

A variety of animal viruses with positive-stranded RNA genomes contain internal ribosome entry sites (IRESs) in their 5′ untranslated region (5′-UTR) [1], [2]. These IRES elements are highly structured and are involved in ribosome recruitment to promote viral mRNA translation. IRESs have been classified according to their phylogenetic origin, secondary structure and functionality. Thus, four major classes of IRESs from picornaviruses, flaviviruses, dicistroviruses and retroviruses have been defined. In addition, picornavirus IRESs have been divided into at least four types or classes. Poliovirus (PV) and human rhinovirus (HRV) IRESs are representative members of class I, while encephalomyocarditis virus (EMC) and foot-and-mouth disease virus (FMDV) IRESs belong to class II. Hepatitis A virus (HAV) IRES has been grouped in class III and, finally, porcine Teschovirus-1 IRES with similarities to hepatitis C virus (HCV) is a representative member of class IV. In addition to differences in the length and structure of these elements, they exhibit different requirements for initiation factors during translation. Protein synthesis directed by all picornavirus mRNAs, with the exception of HAV mRNA, takes place efficiently when eIF4G is cleaved by picornavirus proteases. Thus, translation driven by EMCV and PV IRESs do not require eIF4E or intact eIF4F complex to initiate protein synthesis [3], whereas HAV IRES depends on eIF4F including eIF4E [4], [5], [6]. In fact, the requirement for eIF4E and intact eIF4F complex of HAV IRES constituted one major characteristic to justify placing it in a different group to the other picornaviruses.

The initial report by Whetter et al. (1994) examined translation of monocistronic and dicistronic mRNAs bearing the HAV IRES in monkey kidney cells permissive for HAV, which expressed the T7 RNA polymerase. Protein synthesis directed by these mRNAs was very inefficient and severely inhibited by co-expression of PV 2A^pro^. Subsequent *in vitro* experiments using RRL revealed that cleavage of eIF4G by HRV 2A^pro^ or FMDV L^pro^ strongly reduced HAV IRES-directed translation [4], [7]. This inhibition was rescued by addition of eIF4F, supporting the idea that HAV IRES required intact eIF4G to direct translation. Similar conclusions were reported, describing that inhibition of eIF4E by cap analogous or the presence of 4E-BP blocked HAV IRES-driven translation in RRLs [8]. Apart from these differences in the requirement of eIF4G between HAV and other picornavirus IRESs [9], translation directed by HAV exhibits other features. Thus, cleavage of poly (A)-binding protein (PABP) and polypyrimidine tract-binding protein (PTB) by HAV 3C^pro^ blocks translation of its cognate mRNA [10], [11]. In addition, La autoantigen blocks HAV IRES [12] in contrast to the evidence that this RNA binding protein is a trans-acting factor on PV translation [13].

Recently, we found that translation of different picornavirus mRNAs can take place when eIF2α becomes phosphorylated at late times of infection [14]. In this sense, a dual mechanism is responsible for picornavirus mRNA translation. At early times of infection picornavirus mRNA is translated following a canonical mechanism that employs intact eIF4G and active eIF2, whereas at late times inactivation of eIF2 does not abrogate viral protein synthesis [14]. Moreover, synthesis of PV 2A^pro^ at high levels in culture cells makes translation of mRNAs containing EMCV or PV IRESs independent of eIF2 [15]. Therefore, the presence of PV 2A^pro^ and the cleavage of eIF4G change the mode of initiation of protein synthesis to an eIF2-independent mechanism. The suggestion that cleavage of eIF5B by PV 3C^pro^ renders eIF2-less translation of PV mRNA [16], was not supported by the demonstration that, apart from PV 2A^pro^, none of the PV non-structural proteins provided eIF2-independence for picornavirus IRES-directed protein synthesis [15]. In view of these findings, we decided to analyze the mechanism of translation directed by HAV IRES in the presence of high levels of picornavirus proteases. Surprisingly, PV 2A^pro^ and FMDV L^pro^ exhibit opposing effects on HAV translation. In accord with previous findings, PV 2A^pro^ strongly inhibited HAV IRES-driven translation [6], while FMDV L^pro^ enhanced this translation by several fold. These findings illustrate that contrary to previous ideas, HAV IRES can efficiently direct translation when eIF4G has become cleaved. Under these conditions, HAV translation can occur when eIF2α is phosphorylated.

## Materials and Methods

### Cell Cultures

Huh7-T7 cells (Human Hepatoma) [17] and Baby Hamster Kidney (clon BSR-T7/5, designated as BHK-T7) [18] were used in this work. Both cell types constitutively express the T7 RNA polymerase. Huh7-T7 cells were kindly provided by R. Bartenschlager (University of Heidelberg, Germany). Cells were grown at 37°C in Dulbecco’s Modified Eagle’s Medium (DMEM) supplemented with 10% or 5% fetal calf serum (FCS) and non-essential amino acids. BHK-T7 cells were additionally incubated with Geneticin G418 (Sigma) on every third passage at a final concentration of 2 mg/ml. For Huh7-T7 cells the medium was supplemented with 5 µM Zeocin.

### Plasmids and Transfections

The plasmid encoding HAV(IRES)-luc has been described previously [11], [19]. The construct pTM1-luc has also been already described [20]. pTM1 bears the EMCV IRES element before the corresponding gene. Plasmid T7 Rluc ΔEMC IGR-Fluc (pIGR CrPV-luc) was kindly provided by P. Sarnow (Standford University, USA). Plasmid pFMDV-L was kindly provided by G. Belsham (Technical University of Denmark, Denmark). The different plasmids and mRNAs employed in this work are listed in Table 1. Huh7-T7 and BHK-T7 cells were transfected using Lipofectamine 2000 (Invitrogen). Cells were transfected or co-transfected with the plasmids as indicated in each experiment or with *in vitro* transcribed mRNA. These plasmids or RNAs were added along with 2 µl lipofectamine per well in Opti-mem medium (Invitrogen) and incubated at 37°C for 3 h in the case of the Huh7-T7 cells and 2 h for BHK-T7 cells. The lipofectamine was then removed and the cells were supplemented with fresh medium containing 10% or 5% FCS, respectively.

**Table 1:** Plasmids used in this study.

Plasmid	Description	mRNA
**pTM1-2A**	Plasmid containing *PV 2A* gene after EMCV IRES	EMC(IRES)-2A
**pTM1-L**	Plasmid containing *FMDV Lb* gene after EMCV IRES	EMC(IRES)-L
**pFMDV-L**	Plasmid containing *FMDV L* gene after FMDV IRES	FMDV(IRES)-L
**pTM1-2C**	Plasmid containing *PV 2C* gene after EMCV IRES	EMC(IRES)-2C
**pHAV-luc**	Plasmid containing *luc* gene after HAV IRES	HAV(IRES)-luc
**pIGR CrPV-luc**	Plasmid containing *luc* gene after IGR IRES	CrPV(IRES)-luc

### *In vitro* Transcription and Translation

pHAV-luc, pTM1-2C and pTM1-L were linearized prior to *in vitro* transcription with T7 RNA polymerase (BioLabs) according to the manufacturer’s instructions. *In vitro* translation was carried out in RRL (Promega). To ensure the cleavage of eIF4GI, the lysates were pre-incubated at 30°C for 1 h in the case of EMC(IRES)-L mRNA or for 20 min with the purified protein FMDV L^pro^. Extracts were then treated with 0.5 µg/ml poly(I:C) (PharmaciaBiotech) for 30 min to induce phosphorylation of eIF2α. Subsequently, 100 ng of different mRNAs were added and incubated for 1 h at 30°C. Protein synthesis was estimated by measuring luc activity and by Western blot to analyze the eIF4GI cleavage.

### Inhibitor Treatments and Analysis of Protein Synthesis by Radioactive Labelling

BHK-T7 cells were transfected or co-transfected with the plasmids indicated in each experiment. At 2 hpt, cells were pre-treated with 200 µM sodium arsenite (Ars) (Riedel-de Haën) for 15 min at 37°C, or left untreated. Next, proteins were radiolabelled for 45 min with [^35^S]Met/Cys (Promix; Amersham Pharmacia) in methionine/cysteine-free DMEM in the presence or absence of 200 µM Ars. Finally, cells were collected in sample buffer, boiled for 4 min and analysed by SDS-PAGE (17.5%) and fluorography. Protein synthesis was quantified by densitometry using a GS-710 calibrated Imaging Densitometer (Bio-Rad). In the case of hippuristanol, Huh7-T7 cells were transfected with the indicated plasmids. Hippuristanol was a generous gift of J. Pelletier (McGill University, Canada). The cells were subsequently preincubated with different concentrations of hippuristanol for 30 min then radiolabelled for 60 min with [^35^S]Met/Cys in methionine/cysteine-free DMEM with the same concentrations of the inhibitor. Finally, the cells were processed as described above.

### Purification of FMDV L^pro^

Active Lb^pro^ (FMDV amino acids 29 to 201) was expressed as described previously [21]. Briefly, *E.coli* BL21 Lys E cells containing the plasmid pet11d/Lb were grown to an OD590 of 0.5. Expression was induced with 0.1 mM IPTG and cells were incubated at 30°C for a further 5 h. Cells were lysed by sonication, cleared by low-speed centrifugation and an ammonium sulphate cut of 40–80% made. The pellet was resuspended in buffer A (50 mM Tris-HCl, pH 8.0, 50 mM NaCl, 5 mM DTT, 1 mM EDTA, 5% glycerol), dialysed and loaded onto a 10/10 MonoQ column. Lb^pro^ fractions eluted at around 300 mM NaCl. These were pooled and further fractionated on a superdex 75 Hiload 26/60 column. Lb^pro^ containing fractions were identified, pooled and stored in buffer A containing 50% glycerol at −80°C. Typical yields were between 5 and 7 mg of Lb^pro^ per liter of culture.

### Western Blotting

Transfected cells were collected in sample buffer, boiled and analyzed by SDS-PAGE. After electrophoresis, proteins were transferred to a nitrocellulose membrane as described previously [22]. To detect eIF4GI, rabbit antibodies against the N-terminal and C-terminal portion of this protein [23] were used at 1∶1000 dilution. Polyclonal rabbit antibodies against eIF2α (Santa Cruz biotechnologies) and phosphorylated eIF2α (Cell Signaling) were used at a 1∶1000 dilution. Rabbit antisera were raised against firefly luciferase (Promega). Incubation with primary antibodies was performed for 2 h at room temperature, except for phosphorylated eIF2α, which was incubated overnight at 4°C. Next, the membrane was washed three times with PBS containing 0.2% Tween-20 and incubated for 1 h with horseradish peroxidase-conjugated anti-rabbit IgG antibodies (Amersham) at a 1∶5000 dilution. After washing three times, protein bands were visualized with the ECL detection system (Amersham).

### Measurement of Luciferase Activity

Cells were recovered in a buffer containing 25 mM-glycylglycine (pH 7.8), 0.5% Triton X-100 and 1 mM dithiothreitol. Luc activity was determined using *luciferase assay system* (Promega) and Monolight 2010 apparatus (Analytical Luminescence Laboratory) as described previously [24], [25].

## Results

### Opposite Effects of PV 2A^pro^ or FMDV L^pro^ on HAV IRES-driven Translation

**Figure 1:**
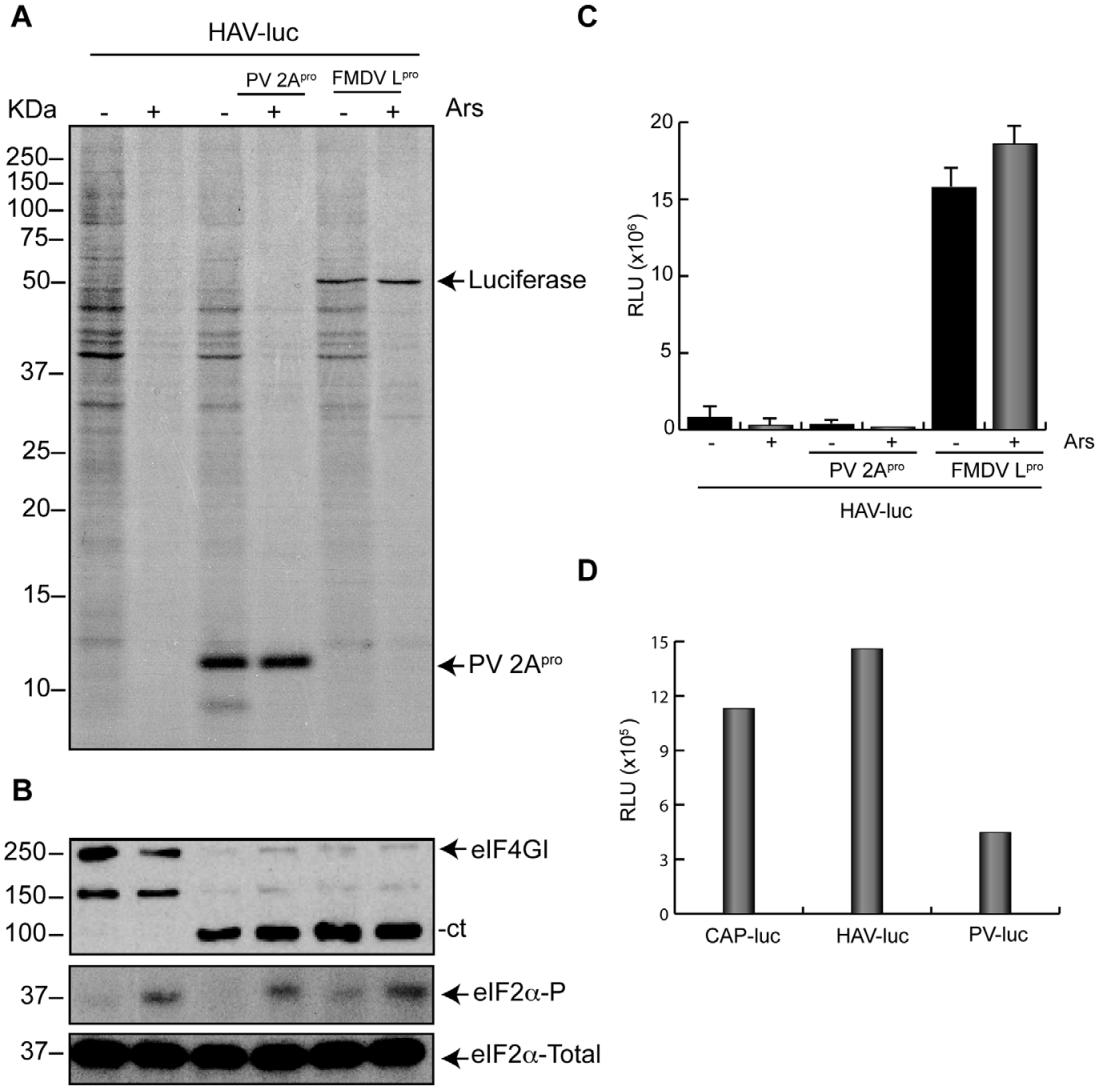
HAV IRES translation in BHK cells after cleavage of eIF4G. A) BHK-T7 cells were transfected or co-transfected for 2 h with 1 µg plasmid encoding HAV(IRES)-luc alone or in presence of 1 µg pTM1-2A or pFMDV-L, respectively. After 2 hpt, cells were treated with 200 µM Ars for 15 min and then metabolically labeled with 0.2 µCi per well [^35^S]Met/Cys in presence (+) or absence (−) of Ars for 45 min. Finally, cells were processed by SDS-PAGE, fluorography and autoradiography. B) The same samples were used to analyze eIF4GI, eIF2α phosphorylation and total eIF2α by western blot using specific antibodies as detailed in Materials and Methods. C) BHK-T7 cells were transfected under the conditions described above. Cells were then collected and processed to assay for luc activity as described in Materials and Methods. The bars represent the luc activity in presence (+) or absence (−) of Ars. The RLUs values obtained were as follows: pHAV-luc in absence (−) or presence (+) of Ars were 3.9×10^5^ and 1.8×10^5^, respectively. pHAV-luc co-transfected with pTM1-2A (−) or (+) Ars were 0.2×10^5^ and 0.1×10^5^, respectively, and finally pHAV-luc co-transfected with pFMDV-L (−) or (+) Ars were 25×10^5^ and 17×10^5^, respectively. Error bars indicate standard deviation (SD). D) BHK-T7 cells were transfected with cap-luc, HAV(IRES)-luc or PV(IRES)-luc mRNAs. At 2 hpt cells were collected and luc activity was measured. The RLUs values obtained were as follows: cap-luc: 1.13×10^6^; HAV(IRES)-luc:1.46×10^6^ and PV(IRES)-luc: 0.44×10^6^.

Several reports have established that HAV IRES-driven translation is abrogated by PV 2A^pro^ or FMDV L^pro^, both in culture cells and in cell free systems [4], [5], [6]. This abrogation is due to the bisection of eIF4G by these proteases, since addition of intact eIF4F complex restores this inhibition. Therefore, HAV IRES seems to be an exception among the other picornavirus IRES analyzed, as regards its requirement for intact eIF4G. This finding together with other differences in IRES structure provided the rationale for classification of the HAV IRES in the type III group [1]. Recently, using the system described in our previous work we found that PV 2A^pro^ had the ability to modify the mechanism of initiation of PV- or EMCV IRESs-directed translation, as regards their requirement for active eIF2 [15]. This system used a BHK cell line that stably expresses T7 RNA polymerase (BHK-T7). After co-transfection of BHK-T7 cells with plasmids encoding luciferase (luc) and PV 2A^pro^ or FMDV L^pro^, there was an efficient expression of luc since these plasmids bear an IRES element under a T7 promoter. Thus, we wanted to test the effects of PV 2A^pro^ or FMDV L^pro^ on translation directed by HAV IRES. Initially, BHK-T7 cells were transfected for 2 h with a plasmid bearing the HAV IRES followed by luc gene and co-transfected with pTM1-2A or pFMDV-L. In addition, to analyze the participation of eIF2 in HAV-driven translation, cells were treated with Ars. This compound induces the activation of the protein kinase HRI that phosphorylates eIF2α [8], [26], [27], [28]. Therefore, at 2 hpt, cells were pre-treated with Ars for 15 min and then radiolabeled by incubating with [^35^S]Met/Cys from 2–3 hpt in presence (+) or absence (−) of 200 µM Ars (Fig. 1A). These same samples were also analyzed by Western blotting to detect eIF4G or eIF2 (Fig. 1B). Synthesis of luc in this system can be detected by estimating luc activity.The amount of luc synthesized after transfection with pHAV-luc is much lower than that obtained by transfection of pTM1-luc, which is about 20–50 fold higher (results not shown). Therefore, under these conditions, luc detection by radiolabeling is not observed (Fig. 1A). In agreement with previous results, co-transfection of pHAV-luc with pTM1-2A strongly blocks the synthesis of luc. In this case, the synthesis of PV 2A^pro^ is detected by SDS-PAGE of the radiolabeled proteins (Fig. 1A), as well as by cleavage of eIF4G (Fig. 1B, upper panel). To our surprise, co-transfection of pHAV-luc and pFMDV-L leads to a clear stimulation of luc synthesis. Indeed, high levels of luc synthesis can be detected by radiolabeling (Fig. 1A), even though eIF4G has been substantially cleaved (Fig. 1B, upper panel). This finding provided initial evidence that luc synthesis directed by HAV IRES at low detectable level is actually increased after eIF4G cleavage by FMDV L^pro^. As regards eIF2α, we analyzed both total eIF2 and phosphorylated eIF2α (Fig. 1B). Addition of Ars clearly induces the phosphorylation of eIF2αin control cells and in cells that express the picornavirus protease. Interestingly, the expression of FMDV L^pro^ partially increased the phosphorylation of eIF2α, even in the absence of Ars (Fig. 1B). In previous work, we have found that treatment of BHK-T7 cells with 200 µM Ars induces the phosphorylation of virtually all eIF2α present in cells [14], [15]. In parallel, cells were transfected under the same conditions and were collected at 3 hpt to measure luc activity. Treatment with 200 µM Ars inhibited luc synthesis by about 60% in BHK-T7 cells transfected with pHAV-luc (Fig. 1C). On the other hand, the presence of PV 2A^pro^ inhibited luc synthesis by 95% in presence or absence of Ars. Remarkably, Ars treatment had no effect on luc synthesis in the presence of FMDV L^pro^ (Fig. 1C). In this case, FMDV L^pro^ estimulated luc synthesis by 6.4fold in absence of Ars and the estimulation was 9.4fold in its presence. This finding supports the idea that translation directed by HAV IRES can occur not only when eIF4G has been cleaved, but also in the absence of active eIF2. For comparative purposes cells were transfected with different mRNAs, in order to analyze their translatability. Thus, cells were transfected with cap-luc, HAV(IRES)-luc and PV(IRES)-luc mRNAs and luc synthesis was estimated after 2 h. As observed in Figure 1D, the level of translation of HAV(IRES)-luc mRNA is similar to that found with cap-luc and even higher than that obtained with PV(IRES)-luc mRNA.

**Figure 2:**
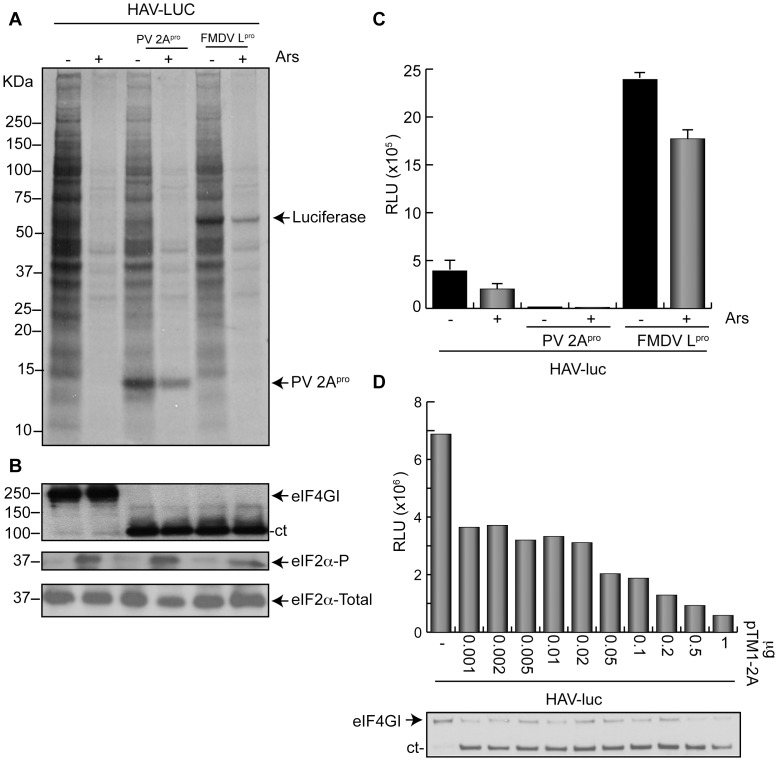
HAV IRES translation in presence of cleaved eIF4G in Huh7-T7 cells. A) Huh7-T7 cells were transfected or co-transfected for 3 h with 1 µg plasmid encoding HAV(IRES)-luc alone or in presence of 1 µg pTM1-2A or pFMDV-L, respectively. After 2 hpt, cells were treated with 200 µM Ars for 15 min and then metabolically labeled with 0.2 µCi per well[^35^S]Met/Cys in presence (+) or absence (−) of Ars for 45 min. Finally, cells were processed by SDS-PAGE, fluorography and autoradiography. B) The same samples were used to analyze eIF4GI, eIF2α phosphorylation and total eIF2α by western blot. C) Huh7-T7 cells were transfected under the conditions described above. Cells were then recovered and processed to assay for luc activity as described in Materials and Methods. The bars represent the luc activity in presence (+) or absence (−) of Ars. The RLUs values obtained were as follows: pHAV-luc in absence (−) or presence (+) of Ars were 4.3×10^5^ and 1.8×10^5^, respectively. pHAV-luc co-transfected with pTM1-2A (−) or (+) Ars were 0.3×10^5^ and 0.2×10^5^, respectively, and finally pHAV-luc co-transfected with pFMDV-L (−) or (+) Ars were 23.4×10^5^ and 17.3×10^5^, respectively. Error bars indicate SD. D) Huh7-T7 cells were transfected with 1 µg plasmid pHAV-luc alone or with increasing concentrations of plasmid pTM1-2A for 3 h. After 3 hpt, cells were recovered and processed to measure luc activity. Values obtained are represented in the graph (upper panel). The same samples were used to analyze eIF4GI cleavage (lower panel).

Since the natural hosts for HAV replication are liver cells, we tested HAV (IRES)-luc mRNA translation in the human hepatoma cell line that stably expresses T7 RNA polymerase (Huh7-T7 cells). Analysis of protein synthesis by SDS-PAGE of cells transfected with pHAV-luc and pTM1-2A or pFMDV-L shows that synthesis of luc is only apparent when FMDV L^pro^ is present (Fig. 2A). When Huh7-T7 cells were transfected with pTM1-2A, the synthesis of this protease was clearly apparent, but no luc synthesis was detected. Cleavage of eIF4G was found when PV 2A^pro^ or FMDV L^pro^ were present, as analyzed by western blotting (Fig. 2B). Treatment of these cells with 200 µM Ars leads to a substantial inhibition of cellular protein synthesis, however, synthesis of PV 2A^pro^ was more resistant to this inhibition, as well as the synthesis of luc when FMDV L^pro^ was present (Fig. 2A). Indeed, phosphorylation of eIF2α took place when cells were treated with Ars (Fig. 2B, middle panel).The synthesis of luc in this system was also tested by measuring luc activity after transfection with pHAV-luc (Fig. 2C). The results obtained were similar to those found with BHK-T7 cells (Fig. 1). In agreement with the above results, co-expression of PV 2A^pro^ blocks HAV(IRES)-luc mRNA translation, but luc synthesis was clearly stimulated by the co-expression of FMDV L^pro^. To further assess that PV 2A^pro^ was inhibitory for HAV IRES-driven translation and also to analyze if there is a correlation between the protease activity and this inhibition, a concentration curve of pTM1-2A on luc synthesis was carried out. Figure 2D shows that increasing concentrations of pTM1-2A are inhibitory for luc synthesis after co-transfection with pHAV-luc. Strikingly, even the transfection of very low concentrations of pTM1-2A were inhibitory for luc synthesis in this system, suggesting that the entry of a few copies of this plasmid into cells leads to its efficient transcription, giving rise to PV 2A^pro^ that is able to partially cleave eIF4G (Fig. 2D, lower panel). Taken together these findings indicate that PV 2A^pro^ and FMDV L^pro^ exhibit opposite effects on translation directed by HAV IRES. Moreover, this translation may occur when eIF4G has been cleaved by FMDV L^pro^ and even when eIF2α has been phosphorylated.

### Translation of HAV(IRES)-luc mRNA after eIF4G Cleavage

**Figure 3:**
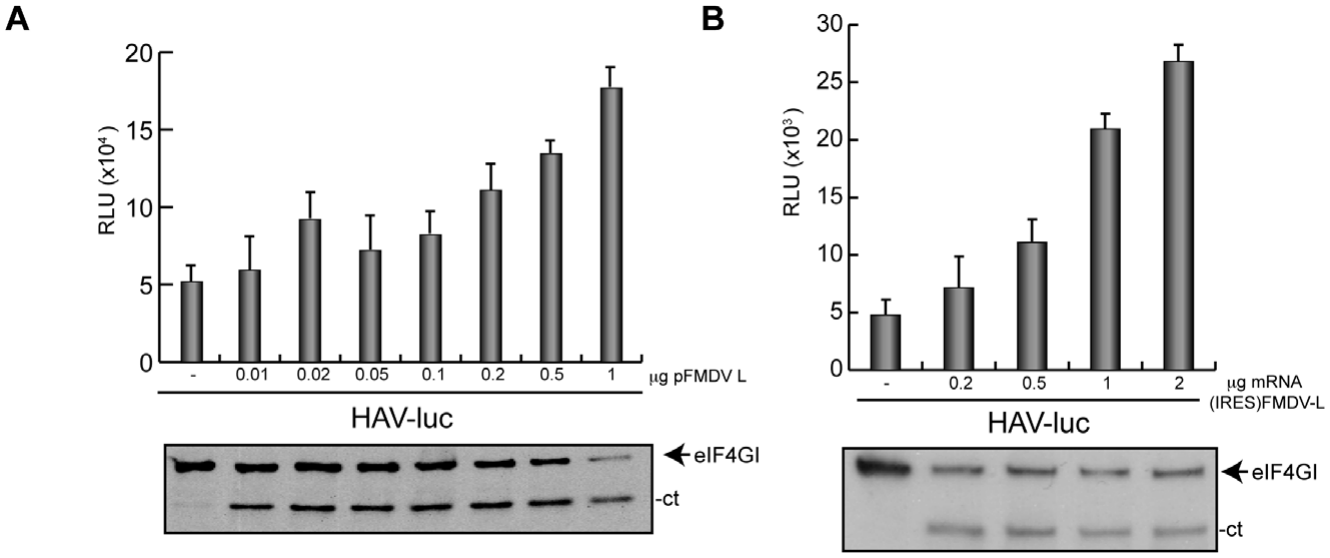
Stimulation of HAV(IRES)-luc mRNA translation is dependent on FMDV L^pro^ concentration. A) Huh7-T7 were transfected for 3 h with 1 µg plasmid encoding HAV(IRES)-luc alone and co-transfected with different concentrations of plasmid encoding FMDV L^pro^. After 3 hpt, cells were harvested, washed in PBS and resuspended in luc buffer. The graph represents luc synthesis in presence of increasing concentrations of pFMDV-L (upper panel). eIF4GI cleavage was analyzed by western blot (lower panel). B) pFMDV-L was linearized and transcribed *in vitro*. Huh7-T7 cells were transfected or co-transfected with 1 µg plasmid pHAV-luc alone or with different amounts of FMDV(IRES)-L mRNA. After 3 h in presence of transfection mixture and 3 h in fresh medium, cells were recovered and luc activity was measured and represented in the graph (upper panel). Error bars represent SD. The same samples were employed to analyze eIF4GI cleavage (lower panel).

**Figure 4:**
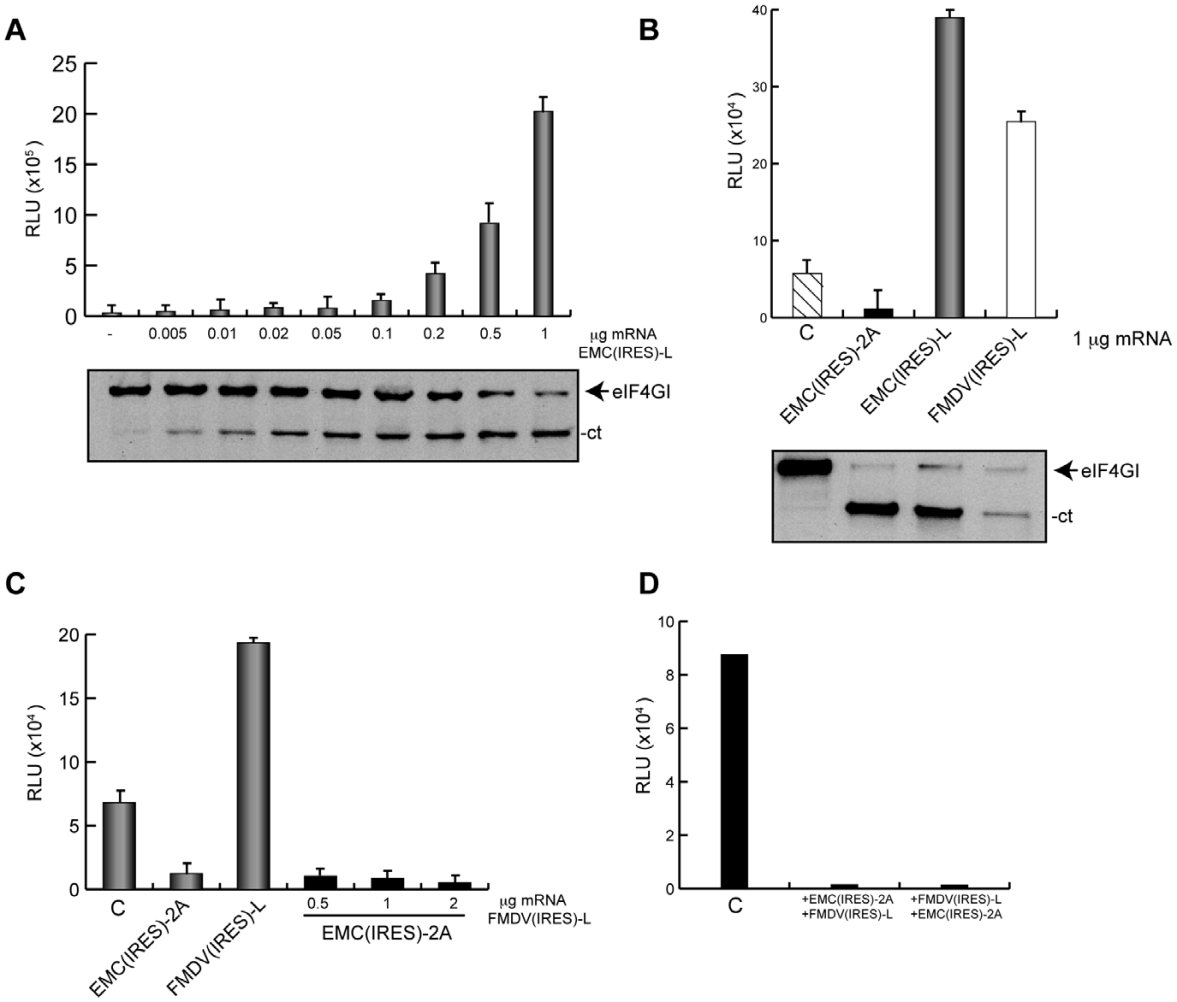
Co-transfection of HAV(IRES)-luc mRNA and FMDV(IRES)-luc mRNA induces a strong stimulation of luc synthesis. A) pTM1-L was linearized and transcribed *in vitro*. EMC(IRES)-L mRNA was obtained. Then, 1 µg pHAV-luc was transfected alone or co-transfected with increasing concentrations of EMC(IRES)-L mRNA for 3 h. At 3 hpt cells were processed as described in Materials and Methods to measure luc activity. Values are represented in the graph (upper panel). Error bars represent SD. The same samples were analyzed by western blot with specific antibodies against eIF4GI (lower panel). B) pHAV-luc was linearized and transcribed *in vitro* to obtain HAV(IRES)-luc mRNA. Then, 1 µg HAV(IRES)-luc mRNA was transfected alone (C) or co-transfected with 1 µg EMC(IRES)-2A mRNA, 1 µg EMC(IRES)-L or 1 µg FMDV(IRES)-L. At 3 hpt cells were processed to measure luc activity. The values of luc activity are indicated on the graph (upper panel). Error bars represent SD. eIF4GI cleavage was analyzed by western blot (lower panel). C) 1 µg HAV(IRES)-luc mRNA was transfected alone (C) or co-transfected with 1 µg EMC(IRES)-2A mRNA or 1 µg FMDV(IRES)-L mRNA for 3 h. Moreover, an mRNA mixture containing 1 µg HAV(IRES)-luc mRNA, 1 µg EMC(IRES)-2A mRNA and increasing concentrations of FMDV(IRES)-L mRNA were transfected for the same time. At 3 hpt cells were collected and luc activity was measured and plotted. Error bars indicate SD. D) Huh7-T7 cells were transfected with 1 µg HAV(IRES)-luc mRNA alone (C) or co-transfected sequentially with both mRNAs, i.e. first 1 µg EMC(IRES)-2A mRNA or 1 µg FMDV(IRES)-L mRNA was added and incubated for 2 h and then cells were transfected with 1 µg FMDV(IRES)-L mRNA or 1 µg EMC(IRES)-2A mRNA, respectively, together with 1 µg HAV(IRES)-luc. After 2 h of incubation cells were collected and luc activity was measured. The values obtained are represented in the graph.

To further assess whether HAV luc mRNA can be translated when eIF4G is cleaved, we have analyzed different expression systems in Huh7-T7 cells. First we assayed luc synthesis in cells transfected with pHAV-luc and co-transfected with increasing concentrations of pFMDV-L. Fig. 3A shows that when the amount of pFMDV-L is increased, there is a partial cleavage of eIF4G and this cleavage is higher when 1 µg pFMDV-L is transfected. Notably, there is an increase in the production of luc by as much as 4 fold. Another system employed to synthesize FMDV L^pro^ was by transfection of the *in vitro* synthesized mRNA. Two different mRNAs were used, FMDV(IRES)-L and EMC(IRES)-L mRNAs. We have observed that the latter of these mRNAs, which contains the IRES from EMCV, directs the synthesis of FMDV L^pro^ even more efficiently than FMDV(IRES)-L mRNA. Therefore, transfection of EMC(IRES)-L mRNA gives rise to a higher stimulation of luc activity and also to a more efficient cleavage of eIF4G. Nevertheless, co-transfection of pHAV-luc with increasing amounts of these mRNAs into Huh7-T7 cells renders eIF4G cleavage and the parallel stimulation of luc synthesis from HAV(IRES)-luc mRNA (Fig. 3B and 4A). These results reinforce the idea that cleavage of eIF4G by FMDV L^pro^ stimulates the translation of HAV(IRES)-luc mRNA.

To rule out the possibility that FMDV L^pro^ specially affected transcription directed by T7 RNA polymerase, cells were co-transfected with both types of mRNAs. To this end, pHAV-luc were linearized and transcribed *in vitro* to obtain HAV(IRES)-luc mRNA. Huh7-T7cells were then transfected with HAV(IRES)-luc mRNA alone or with EMC(IRES)-2A, EMC(IRES)-L or FMDV(IRES)-L mRNAs for 3 h. After transfection, the normal medium is restored and further incubated for 2 h. At this time, cell extracts are collected to measure luc activity. As occurs with DNA transfection, HAV(IRES)-luc mRNA translation is strongly inhibited by EMC(IRES)-2A mRNA but stimulated by both EMC(IRES)-L and FMDV(IRES)-L mRNAs (Fig. 4B). Substantial cleavage of eIF4GI was observed in presence of proteases (Fig. 4B, lower panel).

Our next goal was to analyze the possibility that expression of FMDV L^pro^ might rescue HAV(IRES)-luc mRNA translation in presence of PV 2A^pro^. To assay this, 1 µg HAV(IRES)-luc mRNA was transfected alone or co-transfected with 1 µg EMC(IRES)-2A mRNA or 1 µg FMDV(IRES)-L mRNA. Moreover, HAV(IRES)-luc mRNA was co-transfected with a mixture of 1 µg EMC(IRES)-2A mRNA and different concentrations of FMDV(IRES)-L mRNA (Fig. 4C). At 2 hpt, cells were harvested and lysed to measure luc activity. As expected, the presence of EMC(IRES)-2A diminishes luc synthesis around 8 fold whereas expression of FMDV(IRES)-L mRNA stimulates HAV(IRES)-luc mRNA translation by more than 3 fold. However, when both proteases are present, expression of FMDV(IRES)-L mRNA cannot rescue HAV(IRES)-luc mRNA translation in presence of EMC(IRES)-2A (Fig. 4C). A similar inhibition of HAV(IRES)-luc mRNA by PV 2A^pro^ is observed when both proteases are expressed sequentially, i.e. when PV 2A^pro^ is expressed prior to FMDV L^pro^ or viceversa (Fig. 4D). This result could indicate the possibility that PV 2A^pro^ hydrolyzes some cellular protein necessary for HAV IRES-driven translation.

### Requirement of eIF4A for HAV IRES-driven Translation

**Figure 5:**
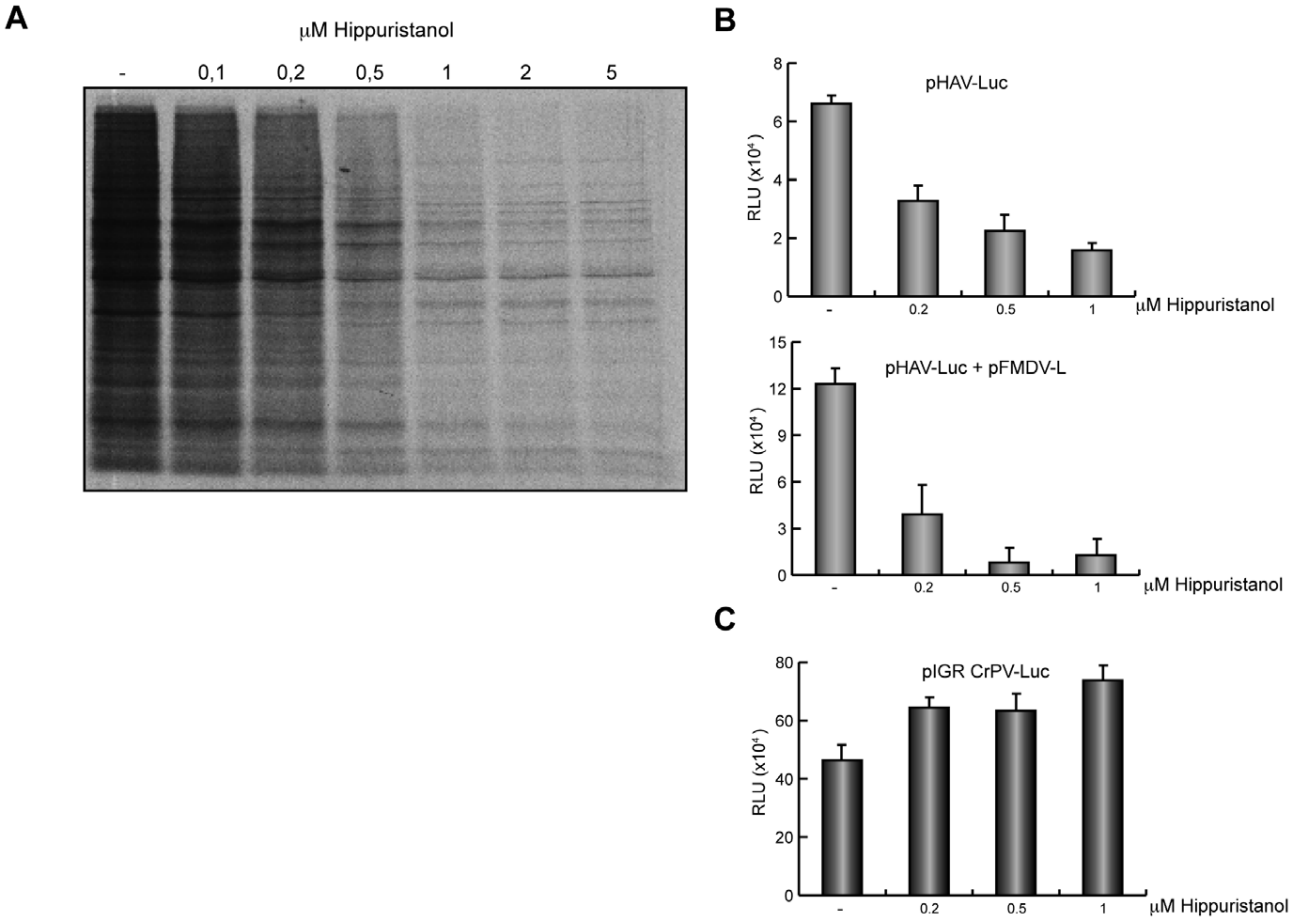
HAV(IRES)-luc mRNA translation is inhibited by hippuristanol. A) Effects of hippuristanol in Huh7-T7 cells. Cells were treated for 30 min with increasing concentrations of hippuristanol and then metabolically labelled with 0.2 µCi per well [^35^S]Met/Cys for 1 h in presence of the inhibitor. Whole-cell extracts were analyzed by SDS-PAGE, fluorography and autoradiography. Dried gels were exposed to X-ray film. B) Huh7-T7 cells were transfected for 3 h with 1 µg plasmid bearing HAV(IRES)-luc in absence (upper panel) or presence (lower panel) of 1 µg pFMDV-L. Then, at 3 hpt, luc activity was measured in presence of increasing concentrations of hippuristanol. C) As control, Huh7-T7 cells were transfected during 3 h with 1 µg pCrPV IGR-luc. After, at 3 hpt, cells were treated with increasing concentrations of hippuristanol for 90 min. Finally, luc activity was measured and the values represented in the graph. Error bars indicate SD.

In recent years, the compound hippuristanol has been used as a selective inhibitor of eIF4A [29], [30]. It is known that both intact eIF4G as well as the carboxy fragment of this factor can interact with HAV IRES [5]. It is also known that eIF4A interacts with this carboxy fragment of eIF4G [31]. For this reason, it was of interest to analyze the participation of eIF4A in the translation of HAV(IRES)-luc mRNA when eIF4G has been cleaved by L^pro^. Addition of different concentrations of hippuristanol to Huh7-T7 cells blocks cellular translation (Fig. 5A), as expected for a selective inhibitor of eIF4A. Luc production in cells transfected with pHAV-luc were also strongly blocked by hippuristanol irrespective of the presence of FMDV L^pro^ (Fig. 5B). As control, pIGR CrPV-luc was used, since this mRNA does not use eIF4A for the initiation of its translation (5). As expected, the addition of hippuristanol does not have an inhibitory effect on luc synthesis mediated by this IRES (Fig. 5C). This observation demonstrates that hippuristanol has no deleterious effects on other steps of translation apart from initiation. In conclusion, these findings indicate that eIF4A perhaps bound to the eIF4G carboxy fragment generated by L^pro^ is required for HAV IRES-driven translation. In addition, this result provides indirect evidence for the participation of the C-terminal fragment of eIF4G in HAV IRES-driven translation.

### Translation of HAV(IRES)-luc mRNA in Cell Free Systems

**Figure 6:**
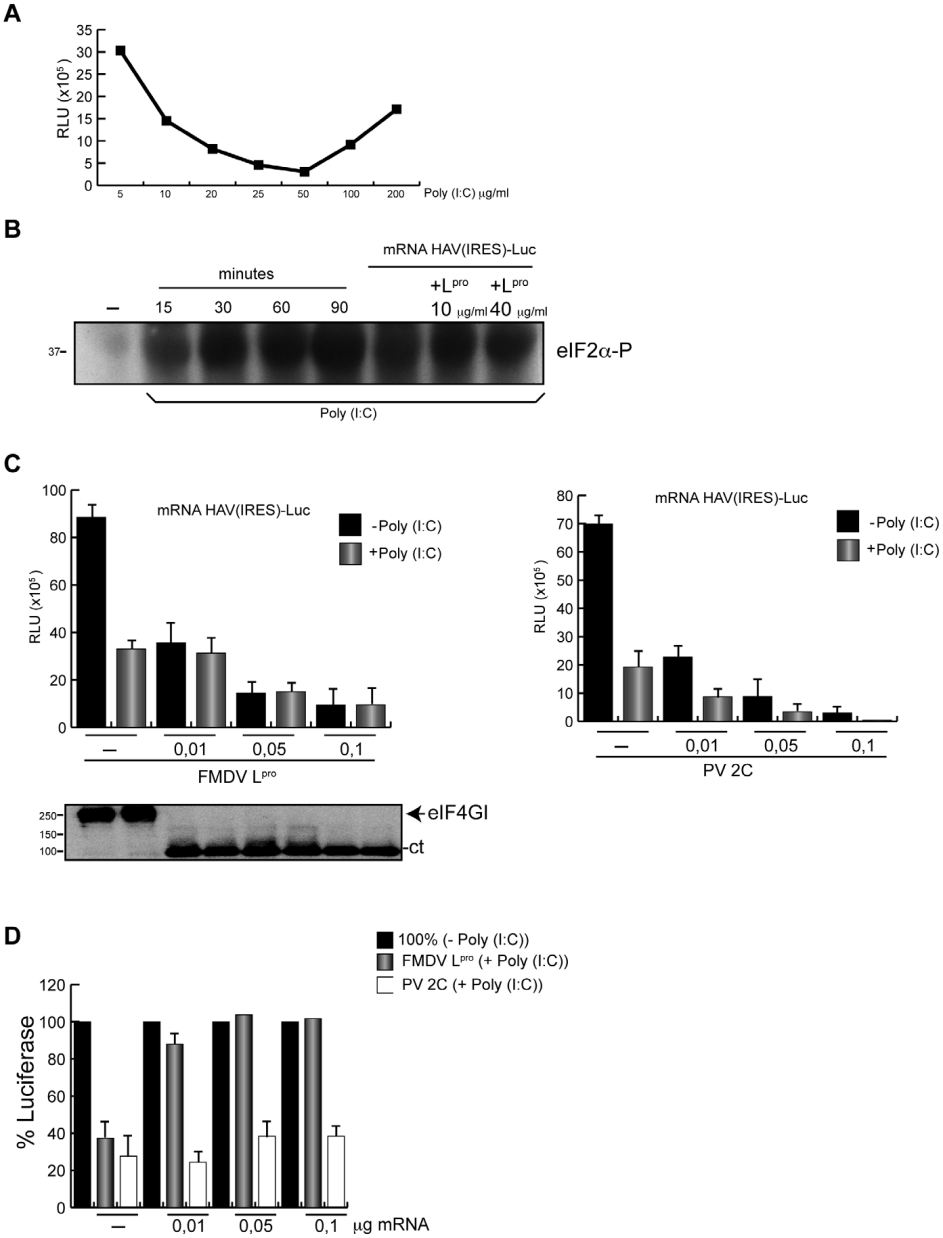
HAV(IRES)-luc mRNA translation in cell free systems. A) RRL were incubated with increasing concentrations of poly(I:C) for 30 min at 30°C. After, cap-luc mRNA was added and incubated for 1 h at the same temperature. Then luc activity was measured. The values obtained are represented in the graph. B) RRL were incubated at 30°C for different time periods with 50 ng poly(I:C). In addition, to analyse the effects of mRNA or L^pro^ on eIF2α phosphorylation, RRL were incubated with the same concentration of poly(I:C) and 100 ng HAV(IRES)-luc mRNA alone or in presence of different amounts of purified L^pro^ for 30 min at the same temperature. Then, eIF2α phosphorylation was analyzed by western blot. C) Plasmids encoding HAV(IRES)-luc, EMC(IRES)-L and EMC(IRES)-2C were linearized and transcribed *in vitro*. The translation reaction was then carried out in RRL at 30°C. First, different concentrations of EMC(IRES)-L mRNA was added for 1 h to ensure eIF4G cleavage. Then, the mixture was incubated with 50 ng poly(I:C) during 30 min and finally 100 ng HAV(IRES)-luc mRNA was added and incubated for 1 h at 30°C. As control, EMC(IRES)-2C mRNA was used. In this case, samples were incubated first with different concentrations of EMC(IRES)-2C mRNA. Then, the mixture was incubated with 50 ng poly(I:C) during 30 min and finally, as above, 100 ng HAV(IRES)-luc mRNA was added and incubated for 1 h at 30°C. The graph represents the RLUs from HAV(IRES)-luc mRNA translation in presence of increasing concentrations of EMC(IRES)-L mRNA (left panel) or EMC(IRES)-2C mRNA (right panel). D) Bars represent the percentage of luc synthesis when eIF2α is phosphorylated in the presence of EMC(IRES)-L mRNA or EMC(IRES)-2C mRNA with respect to values without inhibitor, which are taken as 100%.

Some of the results on the inhibition of translation directed by HAV IRES with picornavirus proteases were obtained in RRL [7], [9]. Therefore, we now decided to use RRL programmed with HAV(IRES)-luc mRNA. The effect of FMDV L^pro^ on this translation was tested using two approaches, one of them provides fresh L^pro^ by the translation of EMC(IRES)-L mRNA whereas the other employs the direct addition of purified L^pro^ to the cell free system. Moreover, we also analyzed the eIF2 requirement for translation of HAV(IRES)-luc mRNA under these conditions using poly(I:C), a compound that induces activation of PKR and eIF2α phosphorylation. Initially, a titration curve of poly(I:C) was carried out in order to obtain the optimal concentration of this compound that blocks translation of a cap-luc mRNA in our system (Fig. 6A). 50 ng/ml poly(I:C) was found to be the optimal concentration that blocked translation in RRL. To analyze eIF2α phosphorylation, RRL treated with this optimal poly(I:C) concentration at different times was tested. Clearly, incubation with this inhibitor leads to phosphorylation of eIF2α, even when FMDV L^pro^ was present (Fig. 6B). The first approach produces newly made L^pro^ by translation of the mRNA encoding this protease under the EMCV IRES sequence. After translation of this mRNA for 60 min, 50 ng poly(I:C) for 30 min was added. Then, HAV(IRES)-luc mRNA was incubated for 1 h. As shown in Fig. 6C, left panel, eIF4G becomes cleaved under these conditions. Surprisingly, inhibition of luc synthesis was observed using this approach (Fig. 6C). Most probably, this inhibition was due to competition of HAV(IRES)-luc mRNA translation by EMC(IRES)-L mRNA. To assay this possibility, a control EMC(IRES)-2C mRNA was tested. This mRNA encodes for PV 2C protein, which is devoid of protease activity under the EMCV IRES. In this case, luc synthesis was also inhibited when the concentration of EMC(IRES)-2C mRNA was increased, suggesting the existence of competition between both mRNAs (Fig. 6C, right panel). Notably, the effect of poly(I:C) was significantly different when EMC(IRES)-L or EMC(IRES)-2C mRNAs were assayed. Indeed, when EMC(IRES)-L mRNA was present no inhibition by poly(I:C) was observed whereas in the case of EMC(IRES)-2C the presence of poly(I:C) led to over 70% inhibition of luc synthesis (Fig. 6D). This result indicates that FMDV L^pro^ can confer translatability to HAV IRES when eIF2α is phosphorylated.

**Figure 7:**
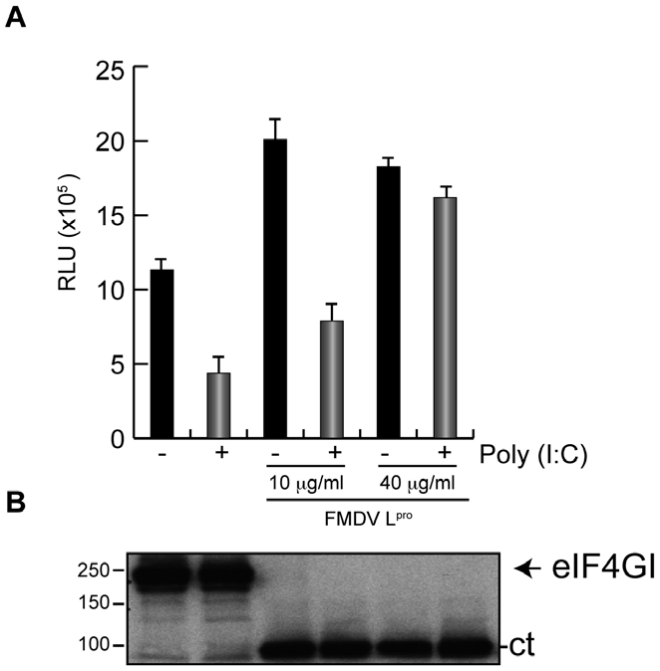
In vitro translation of HAV(IRES)-luc mRNA in presence of purified L^pro^. HAV IRES was tested in RRL in presence of purified protease FMDV L^pro^. First, two different concentrations of protease were added, 10 µg/ml or 40 µg/ml, for 20 min at 30°C. Lysates were then incubated with 50 ng poly(I:C) at the same temperature and,finally, HAV(IRES)-luc mRNA was added and incubated for 1 h at 30°C. Then, aliquots of these samples were processed to measure luc activity (A) and to analyze eIF4GI cleavage (B).

The other approach consisted of direct addition of purified L^pro^ to RRL. After pre-incubation for 20 min with the purified protease, 50 ng poly(I:C) was added and further incubated for 30 min. Then, HAV(IRES)-luc mRNA was added to RRL for 1 h. Clearly, a stimulation of about 3 fold of luc synthesis was found when eIF4G cleavage took place (Fig. 7A and 7B). In conclusion, these findings are in contrast to those previously reported indicating that FMDV L^pro^ blocks translation directed by HAV IRES in RRL [4]. Phosphorylation of eIF2α inhibits HAV(IRES)-luc mRNA translation by around 60%, but no inhibition was found when 40 µM L^pro^ was present (Fig. 7A). We conclude that *in vitro* translation of HAV(IRES)-luc mRNA can take place after eIF4G cleavage by L^pro^ and in presence of phosphorylated eIF2α.

## Discussion

Picornavirus mRNAs contain rather long 5′-UTRs that are highly structured and bear an IRES element. These IRESs drive translation by an initiation mechanism in which ribosomes directly interact with an internal region at or upstream to the initiator AUG_i_ [1], [2]. This mechanism of initiation does not require intact eIF4G, thus, cleavage of this factor by picornavirus proteases does not impair and, in some instances, even stimulates IRES-directed translation [32]. For many years, it has been thought that HAV IRES was an exception to this rule, since cleavage of eIF4G by PV 2A^pro^ or FMDV L^pro^ abrogated translation of mRNAs containing HAV IRES [5], [6], [7]. In addition, the inhibition of eIF4E by 4E-BP1 impairs translation directed by HAV IRES [8], but surprisingly, these authors reported that HAV IRES can be translated in presence of the carboxy fragment of eIF4G in RRL depleted of this factor. One possible explanation for this result is that high concentrations of the carboxy fragment of eIF4G can restore translation of capped mRNAs in eIF4G-depleted RRL [33]. In the present work we provide evidence that HAV IRES translation can occur when eIF4G is cleaved by FMDV L^pro^ and thus HAV IRES does not represent an exception to the rest of picornavirus IRES functioning in this regard. We can now conclude that translation directed by all picornavirus IRESs tested can occur when eIF4G has been cleaved. The divergence in the functioning of the different picornavirus IRESs analyzed may be lower than previously suspected [1]. Perhaps, the classification of HAV IRES in a different group (type III) can now be reconsidered. Although we do not know the reason why our results are so different from those previously reported, we believe that our findings with FMDV L^pro^ are very clear. Thus, this protease not only does not block HAV IRES-luc mRNA, but it stimulates its translation by several fold when eIF4G has been virtually totally cleaved. Previous works testing the requirement for intact eIF4F complex to translate HAV IRES mRNAs mostly used dicistronic mRNAs, bearing a capped structure in the first cistron and followed by the HAV IRES. In these works FMDV L^pro^ strongly inhibited (over 80%) translation driven by HAV IRES [4], [16]. Perhaps, the use of dicistronic mRNAs have provided misleading results. However, in some of these studies, monocistronic mRNAs bearing the HAV IRES were also analyzed. In our present work we have used monocistronic mRNAs, as this approach is, in our opinion, more physiological than the use of dicistronic mRNAs. Although these mRNAs have been very useful for providing evidence of internal initiation, monocistronic mRNAs should be a better option for understanding the mechanism of IRES functioning [2], [34]. Another possibility to account for the discrepancies between previous reports and our present observations is that the amount of FMDV L^pro^ employed was too high or even it contained an inhibitor unrelated to the protease itself.

It is puzzling to observe that PV 2A^pro^ and FMDV L^pro^ exhibit opposite effects as regards to the translation of HAV(IRES)-luc mRNA. One obvious possibility is that apart from eIF4G, PV 2A^pro^ cleaves a factor that is necessary for HAV IRES-driven translation. However, addition of purified eIF4F complex restores the inhibition of PV 2A^pro^ on HAV translation [4]. Another possibility is that the carboxy fragments of eIF4G generated by its protease are not exactly similar. Thus, PV 2A^pro^ cleaves eIF4G at position 681–682, which is located seven residues upstream from the position used by FMDV L^pro^, 674–675 [32]. Thus, the eIF4G carboxy fragment generated by FMDV L^pro^ is seven residues longer than the one originated by PV 2A^pro^. However, we believe that this possibility is very unlikely and most probably PV 2A^pro^ cleaves a factor that is necessary to translate HAV(IRES)-luc mRNA. In agreement with this idea, the simultaneous transfection of mRNAs encoding PV 2A^pro^ and FMDV L^pro^ strongly blocks HAV IRES. Our findings also indicate that the C-terminal fragment of eIF4G generated by FMDV L^pro^ is employed to translate HAV IRES, since this fragment bound to eIF4A is necessary to translate mRNAs bearing picornavirus IRESs [31], [35]. In this regard, hippuristanol, a selective inhibitor of eIF4A, blocks translation directed by picornavirus mRNAs [29], [36]. As demonstrated in this work, eIF4A participates in protein synthesis directed by HAV IRES when eIF4G is intact or even after its cleavage.

Efforts to understand the mechanism by which picornavirus mRNAs are translated have been made over the past four decades. It is surprising that there are still novel and unsuspected findings about the mechanism of initiation of protein synthesis on mRNAs bearing picornavirus IRESs. In this respect, we recently found that PV 2A^pro^ made translation of mRNAs containing PV or EMCV IRESs independent of eIF2 [14], [15]. Since the early days of picornavirus translation, it was thought that this mRNA required eIF2 to initiate translation [3], [37], [38]. Now, we provide evidence that another picornavirus protease, FMDV L^pro^, modifies the requirement for eIF2 to translate HAV(IRES)-luc mRNA. Protein synthesis directed by this mRNA is inhibited by Ars in culture cells or by poly(I:C) treatment of RRL, but this inhibition is not observed if L^pro^ is present. Therefore, translation directed by HAV IRES can occur not only when eIF4G has been cleaved by FMDV L^pro^, but also when eIF2α has been inactivated by phosphorylation. To achieve eIF2 independence for this translation, high levels of FMDV L^pro^ are necessary. Thus, low concentrations of this protease that lead to cleavage of eIF4G do not render translation independent of eIF2. This finding indicates that the simple cleavage of eIF4G by FMDV L^pro^ does not suffice to confer eIF2-independent translatability of HAV(IRES)-luc. This result is in good agreement with our previous observations, demonstrating that high levels of PV 2A^pro^ are necessary for eIF2-independent translation directed by EMC or PV IRESs [15]. In this regard, translation of HAV IRES without intact eIF4G and eIF2 is similar to picornavirus IRES type I (PV) or type II (EMC) [15]. Our present results can serve to promote further research on the mechanism of picornavirus mRNA translation. Future studies could aim to understand the exact mechanism by which the initiation of picornavirus mRNA translation occurs when PV 2A^pro^ or FMDV L^pro^ is present.
